# A Universal, Genomewide GuideFinder for CRISPR/Cas9 Targeting in Microbial Genomes

**DOI:** 10.1128/mSphere.00086-20

**Published:** 2020-02-12

**Authors:** Michelle Spoto, Changhui Guan, Elizabeth Fleming, Julia Oh

**Affiliations:** a The Jackson Laboratory for Genomic Medicine, Farmington, Connecticut, USA; University of Georgia

**Keywords:** CRISPR, Cas9, genomewide knockdown, microbiome

## Abstract

The CRISPR/Cas system has significant potential to facilitate gene editing in a variety of bacterial species. CRISPR interference (CRISPRi) and CRISPR activation (CRISPRa) represent modifications of the CRISPR/Cas9 system utilizing a catalytically inactive Cas9 protein for transcription repression and activation, respectively. While CRISPRi and CRISPRa have tremendous potential to systematically investigate gene function in bacteria, few programs are specifically tailored to identify guides in draft bacterial genomes genomewide. Furthermore, few programs offer open-source code with flexible design parameters for bacterial targeting. To address these limitations, we created GuideFinder, a customizable, user-friendly program that can design guides for any annotated bacterial genome. GuideFinder designs guides from NGG protospacer-adjacent motif (PAM) sites for any number of genes by the use of an annotated genome and FASTA file input by the user. Guides are filtered according to user-defined design parameters and removed if they contain any off-target matches. Iteration with lowered parameter thresholds allows the program to design guides for genes that did not produce guides with the more stringent parameters, one of several features unique to GuideFinder. GuideFinder can also identify paired guides for targeting multiplicity, whose validity we tested experimentally. GuideFinder has been tested on a variety of diverse bacterial genomes, finding guides for 95% of genes on average. Moreover, guides designed by the program are functionally useful—focusing on CRISPRi as a potential application—as demonstrated by essential gene knockdown in two staphylococcal species. Through the large-scale generation of guides, this open-access software will improve accessibility to CRISPR/Cas studies of a variety of bacterial species.

**IMPORTANCE** With the explosion in our understanding of human and environmental microbial diversity, corresponding efforts to understand gene function in these organisms are strongly needed. CRISPR/Cas9 technology has revolutionized interrogation of gene function in a wide variety of model organisms. Efficient CRISPR guide design is required for systematic gene targeting. However, existing tools are not adapted for the broad needs of microbial targeting, which include extraordinary species and subspecies genetic diversity, the overwhelming majority of which is characterized by draft genomes. In addition, flexibility in guide design parameters is important to consider the wide range of factors that can affect guide efficacy, many of which can be species and strain specific. We designed GuideFinder, a customizable, user-friendly program that addresses the limitations of existing software and that can design guides for any annotated bacterial genome with numerous features that facilitate guide design in a wide variety of microorganisms.

## Abstract (precis)

With the explosion in our understanding of human and environmental microbial diversity, corresponding efforts to understand gene function in these organisms are strongly needed. CRISPR/Cas9 technology has revolutionized interrogation of gene function in a wide variety of model organisms. Efficient CRISPR guide design is required for systematic gene targeting. However, existing tools are not adapted for the broad needs of microbial targeting, which include extraordinary species and subspecies genetic diversity, the overwhelming majority of which is characterized by draft genomes. In addition, flexibility in guide design parameters is important to consider the wide range of factors that can affect guide efficacy, many of which can be species and strain specific. We designed GuideFinder, a customizable, user-friendly program that addresses the limitations of existing software and that can design guides for any annotated bacterial genome with numerous features that facilitate guide design in a wide variety of microorganisms.

## INTRODUCTION

The CRISPR/Cas system represents a considerable development in gene editing technology for a wide variety of organisms. Sequence-specific targeting is possible through interactions between a complementary guide RNA and the target sequence and between the protospacer-adjacent motif (PAM) and the Cas nuclease. At the target sequence, the Cas nuclease induces a double-stranded break which is subsequently repaired by the cell by the use of nonhomologous end-joining (NHEJ), if present. This often results in deleterious insertion or deletion mutations that can disrupt the function of the target gene.

Given the broad activity and efficacy of Cas9, the CRISPR/Cas system has been used to successfully edit genes across a diverse range of species (1–3), but its application to bacterial genome editing has been more limited. For instance, many bacterial species do not possess the machinery to efficiently repair double-stranded breaks, and targeting with CRISPR/Cas is consequently lethal to the cell. Additionally, homologous recombination (HR)-mediated repair requires introduction of a second template either as linear DNA or on a supplemental plasmid. Nevertheless, the CRISPR/Cas9 system has significant potential to facilitate gene-editing in a wide range of microorganisms (4). Moreover, additional tools that do not depend on HR or NHEJ for disrupting gene function have since been developed, including CRISPR interference (CRISPRi) and CRISPR activation (CRISPRa).

CRISPRi and CRISPRa are modifications of the CRISPR/Cas system that employ a catalytically inactive Cas9 protein (dCas9) for targeting (5). In the case of CRISPRi, dCas9 is used for transcriptional repression resulting from sterical blocking of transcription machinery and preventing initiation or elongation, depending on the location of the target sequence (on the promoter or DNA strand). CRISPRi has been recently applied on a genomewide scale to identify essential genes and phage host factors in Escherichia coli (6, 7). Given the ease of CRISPRi, this technology has the power to investigate gene function in a variety of genetically diverse, nonmodel microorganisms on a genomewide scale; thus, flexible programs for high-throughput guide design in draft bacterial are critically needed. CRISPRa works similarly, except it is fused to the omega subunit of RNA polymerase, allowing increased recruitment of the polymerase when targeted to sequences upstream of the −35 box of the promoter (5). While, to our knowledge, CRISPRa has not been systematically applied genomewide in bacteria, it represents an interesting potential application of this emerging technology.

For all systems, the efficiency of targeting and the occurrence of off-target effects elsewhere in the genome are influenced by guide selection. A distance of a guide from a transcription start site (TSS) (8), the GC content (9), the homopolymer content (10), cross-reactivity to similar sequences in the genome, and sequence-specific toxicity (11) have all been shown to affect targeting efficacy. While these guide design constraints are important for efficient targeting, consideration of these multiple factors during guide selection makes manual guide design impractical in large scale. This is of particular importance in, for example, genomewide CRISPRi and CRISPRa studies, which require the design of thousands of guides (12). Note also that additional factors such as transcript/protein half-life and secondary structures that are difficult or impossible to predict *a priori* from genome sequence can also affect targeting, increasing the difficulty of systematic prediction of guides.

Existing tools for guide design are limited in their generalizability to large numbers of diverse microbial genomes, which can differ greatly in GC content, length, and number of repeat regions. Indeed, the majority of single guide RNA (sgRNA) design tools have been developed exclusively for eukaryotes or for a few model organisms (11, 13–16). Other programs possess flexibility with respect to the input genome but are limited by their lower-throughput design (17), absence of user-defined filtering parameters and inability to design paired guides (18), or inability to automatically perform iterations with relaxed design parameters (17, 18). We have created GuideFinder to address these limitations, providing a single, user-friendly R script that is a useful advance over existing algorithms.

## RESULTS AND DISCUSSION

**FIG 1:**
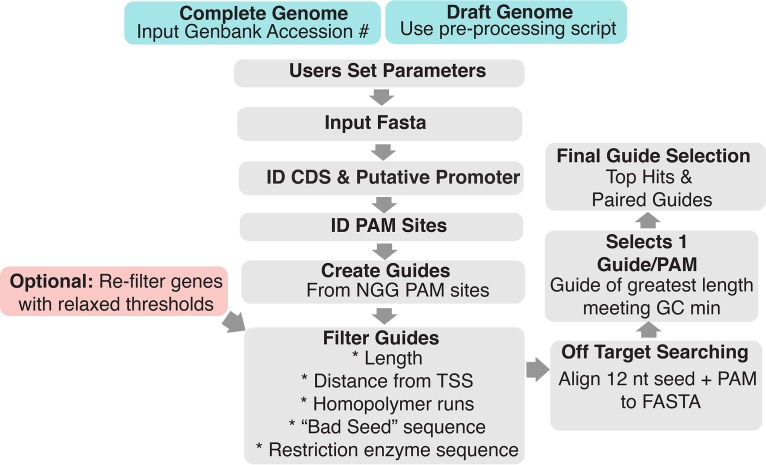
GuideFinder workflow. Users set parameters and input FASTA files. Coding sequence and promoter coordinates are identified and used to obtain sequences. PAM sites are identified, and guides are created and filtered. Off-target searching is conducted using BLAST. The final guide selection step creates a top hits list and a paired guides list. Genes without guides are identified and re-run with relaxed parameters. CDS, coding DNA sequence; PAM, protospacer-adjacent motif; TSS, transcription start site; nt, nucleotide.

Creation and implementation of GuideFinder are described in Materials and Methods and outlined in Fig. 1. Here, we discuss GuideFinder’s key features in the context of existing programs for guide design.

### Draft genome guide design.

Some highly utilized software programs, such as CHOP-CHOP (19), CCTop (14), and CRISPRdirect (20), are suitable only for available complete genomes or perform only off-target searching of reference genomes. However, some species exhibit tremendous within-species variation. One example is that of Staphylococcus epidermidis, whose gene content can differ up to 20% between strains (21, 22). In such cases, the utility of a reference genome for guide design is limited. Additionally, as the number of sequenced strains for a given species increases with decreasing sequencing costs, the importance of tools for guide discovery that can utilize these draft genomes increases. GuideFinder is designed for use with NCBI reference complete or draft genomes (which can be called directly using the genome accession number) or with user-inputted draft genomes.

### User-defined filtering parameters.

While most well-utilized programs have options for some user-defined filtering parameters, no program provides the level of flexibility that GuideFinder affords in combination with the ability to use bacterial draft genomes. Specifically, CCTop allows users to filter by guide length and off-target prediction (14); CHOP-CHOP allows users to filter by guide length, GC content, off-target prediction, self-complementarity, and guide efficiency score (as predicted in mammalian cell lines) (19); and sgRNAcas9 allows users to filter by guide length, off-target prediction, and distance between paired guides (18). Some of these programs elegantly incorporate knowledge about guide efficiency prediction in mammalian cell lines (19). But since none of these programs were developed specifically for targeting in bacterial cells, they lack the functionality to use nonreference genomes (14, 19) or to filter on the basis of guide sequence features that may affect guide efficiency in bacterial cells, such as homopolymer runs of As and Ts and the “bad seed” effect in which the presence of a short, specific nucleotide sequence contained in a targeting guide results in a strong fitness defect (14, 17, 19). GuideFinder allows users to adjust the following filtering parameters either initially or iteratively (allowing the user to relax guide design parameters to increase the number of predicted targets): (i) distance from the transcription start site; (ii) guide length; (iii) GC content; (iv) off-target predictions; (v) sequence features such as homopolymer runs of As/Ts, the “bad seed effect” (sequence toxicity), and restriction enzyme sequence; and (vi) distance between guides, for paired-guide searches (i.e., simultaneous multiguide targeting of a single gene in the same cell).

### Multilocus targeting.

We hypothesized that the efficiency of CRISPRi and CRISPRa can be augmented by targeting multiple loci simultaneously and designed GuideFinder for flexibility for multiguide design. GuideFinder has the ability to design guides that can be used to target different instances of the same gene simultaneously, taking into account the size of the Cas footprint to help prevent steric hindrance of two or more CRISPR/Cas complexes (23), with 100-bp default spacing between guides.

### Iterative guide design.

For genomewide or large-scale guide design, users may want to design guides with stringent filtering parameters on first pass and then, if no guides are identified for a particular gene, relax the parameters to enable recovery of additional guides. GuideFinder automatically identifies genes lacking guides and iteratively searches these genes with relaxed parameters. This unique feature allows users to identify additional guides without compromising the stringency for genes that produce several guides.

### User-friendly design and open-source code.

We acknowledge that CRISPR/Cas targeting in bacterial genomes is an emerging technology. As such, it is likely that new biological findings will refine filtering parameters and guide design rules as the field grows. For example, the importance of the “bad seed” effect was identified in E. coli only recently (11). Thus, it is imperative that GuideFinder can be easily modified to grow with the field as new knowledge about guide design rules for CRISPR/Cas targeting of bacterial genomes emerges. For this reason, GuideFinder is provided as a well-commented Rmarkdown script designed for users with little R programming experience and can also be readily edited to adapt to emerging rules in guide design or for use with alternative PAM sites.

### Scramble guide design.

GuideFinder has the optional ability to design scramble guides—i.e., guides with no match to the input genome—that can act as controls for CRISPRi/a studies. To our knowledge, this is a feature unique to GuideFinder. The ability to generate scramble guides is an important, albeit simple function in GuideFinder that helps to improve access to high-throughput CRISPR studies in bacterial species.

Overall, the GuideFinder program is unique in that it combines all of the following conditions in one program. (i) Reference or draft genomes can be utilized, and off-target searches are performed against the actual genome in use and not a reference genome. (ii) Guides are filtered according to user-defined parameters that are suggested to affect Cas targeting in bacterial genomes. (iii) Guides can be designed for multiguide targeting of a gene. (iv) If too few guides are identified, guide design parameters can be relaxed; iteration through the software produces additional guides. (v) Optionally, scramble/nontargeting guides, which are useful as nontargeting controls, can be identified for the input genome. (vi) GuideFinder has been annotated in detail and was designed for users with little to no programming experience. In addition, the open-source code allows advanced users to make modifications to the program to suit the needs of an emerging field.

### *In silico* and *in vitro* testing of GuideFinder.

GuideFinder is intended to reduce the effort required to design guides targeting genes in any bacterial species, to accommodate both complete and draft genome annotations, and to provide to users the flexibility to adjust the design parameters that may be vital with respect to the organism of interest. GuideFinder was not designed to identify (in general) “better” or more-efficient guides—the identity of such guides is likely highly dependent on the bacterial species or strain of interest, and ideal design parameters suitable for all bacterial species are unlikely to be determined. Rather, GuideFinder is intended to provide to users a flexible program that can design guides using user-defined species or strain-specific parameters (e.g., setting a low guide GC minimum while working with a GC-poor species, filtering guides that contain a known “bad seed” for the organism of interest, or retaining A/T homopolymer runs upon iteration in genes with few guide options) and to be readily adaptable to include new features as the field develops. Although users can elect to design guides for just one gene or just a few genes, if desired, the program is intended to be particularly useful for large-scale guide design. Next, focusing on GuideFinder’s applications for CRISPRi, to investigate these intended uses, we conducted tests *in silico* and *in vitro* to determine (i) the utility of the program across diverse bacterial species and (ii) the ability of the program to design functional guides. We demonstrate its utility in selecting guides genomewide for a diverse set of bacterial species and its ability to select functional guides suitable for gene knockdown.

### Guides for diverse genomes. (i) Testing on complete genomes.

**FIG 2:**
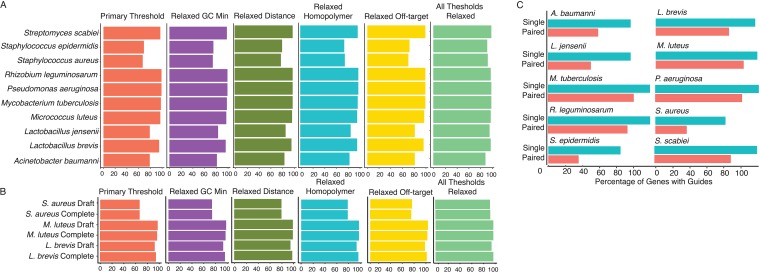
Performing testing on complete and draft genomes *in silico*. (A) Complete genomes. GuideFinder was tested on 10 complete genomes under conditions of primary design constraints with iteration under conditions of relaxed constraints (individually and in combination). (B) Paired versus single guides. The percentages of genes targeted by single guides versus paired guides were compared. (C) Complete genomes versus draft genomes. GuideFinder was tested on 3 draft genomes; the percentage of genes with guides was compared to a complete genome of the same species.

GuideFinder was used to create guides across the genome for a diverse set of 10 complete bacterial genomes (Table 1). These genomes were selected for their diversity in genome size, percentage of gene duplications, and GC content. For each genome, preliminary parameters were set as a GC minimum of 35%, a maximum distance from the TSS of 30%, and a minimum distance between guides of 100 bp, based on the projected footprint of the Cas9 protein (23). These parameters have been utilized previously in our laboratory for successful gene knockdown performed with CRISPRi and thus represent rational design constraints. For each genome, genes that did not produce suitable guide pairs or single guides were identified by the program. These genes were re-run with the following constraints: a GC minimum of 30%, a maximum distance from the TSS of 50%, retention of guides with homopolymers, and relaxed off-target searching. These parameters were relaxed individually and in combination. The GuideFinder program was able to successfully select guides for each of the diverse genomes irrespective of genome size or GC content, but differences in outputs and run time were observed (Fig. 2).

**TABLE 1:** Complete genomes tested^*a*^

Phylum	Organism	Genome size (Mb)	% GC content
*Firmicutes*	Lactobacillus brevis	2.29	45
	Lactobacillus jensenii	1.67	34
	Staphylococcus aureus	2.82	33
	Staphylococcus epidermidis	2.49	32

*Proteobacteria*	Acinetobacter baumannii	4.33	39
	Rhizobium leguminosarum	4.85	60
	Pseudomonas aeruginosa	6.26	66

*Actinobacteria*	Mycobacterium tuberculosis	4.41	66
	Micrococcus luteus	2.5	73
	Streptomyces scabiei	10.41	71

a Ten complete genomes, obtained from NCBI, were selected for their various genome sizes and levels of GC content.

### GC content.

As expected, the genomes with lower (<40%) GC content were less successful in producing usable guides for each gene. For the S. epidermidis, S. aureus, Acinetobacter baumannii, and Lactobacillus jensenii genomes (GC content of 33%, 32%, 39%, and 34%, respectively), the percentages of genes producing guides by the use of the primary filtering thresholds were considerably lower than the average for all 10 genomes (87.5%) at 68%, 67%, 79%, and 79%, respectively. The average for the genomes with >40% GC content was 97.5%. However, for the genomes with low GC content, iteration with lowered parameters was very useful in recovering genes that did not originally produce guides. With each design constraint relaxed in combinations of constraints, the percentages of genes with guides improved to 98%, 93%, 89%, and 96% for S. epidermidis, S. aureus, A. baumannii, and L. jensenii, respectively (Fig. 2A).

### Gene duplications.

We hypothesized that a genome known to contain a high percentage of gene duplications, such as Mycobacterium tuberculosis, would have difficulty producing a large number of usable guides, owing to the high probability of off-target matching. Surprisingly, however, this genome was able to create guides for 98% of the genes using primary thresholds, probably owing to its relatively high GC content (65%).

### Genome size.

Although GuideFinder was run successfully on each of the 10 genomes tested, run times increased with genome size due to the increased number of genes and the subsequently increased number of potential guides (each of which is analyzed for GC content, location, etc.). For example, the program takes approximately 10 min to complete using the S. epidermidis genome (2.49 Mb) but takes approximately 18 h for the largest genome tested, that of Sarcoptes scabiei (10.41 Mb). The genome of S. scabiei is one of the largest known bacterial genomes; thus, we do not expect that this issue will affect most users, but it represents a potential area of improvement for future versions of GuideFinder.

### (ii) Testing on draft genomes.

Three draft genomes were selected to test utility for incomplete genome annotations and were compared to a complete genome annotation of the same species. Draft annotations were obtained from the Pathosystems Resource Integration Center (PATRIC) (24), and whole-genome nucleotide sequences and coding sequences for incomplete genomes were obtained from NCBI. Incomplete genomes were preprocessed with the supplied script to identify gene coordinates. Incomplete genome annotations were successfully used to design guides across the genome for each of the three species tested. In terms of percentages of genes with identified guides and run time, there are no appreciable differences between complete and incomplete genome annotations (Fig. 2B). This result highlights the utility of the program for both types of genome annotation files.

### Essential gene knockdown to validate guides.

**FIG 3:**
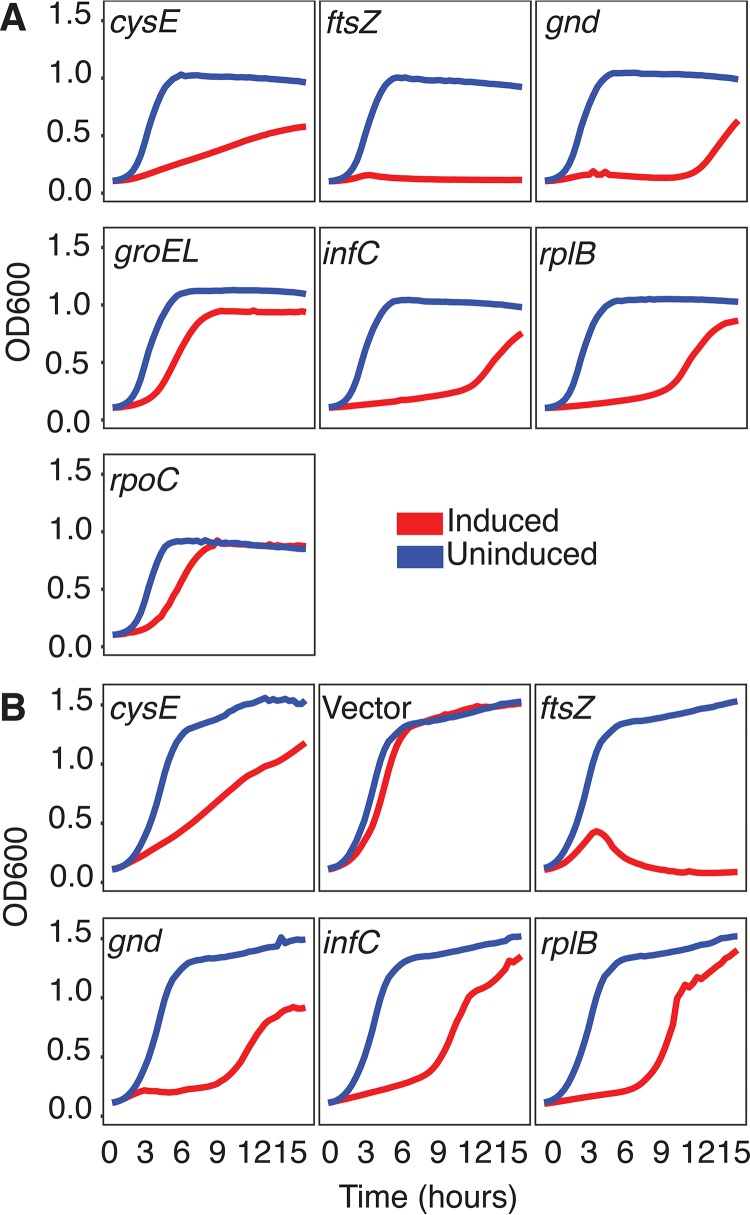
Essential gene knockdown. Essential genes were targeted for knockdown in S. aureus (A) and S. epidermidis (B), and growth curves were created from OD measurements over the course of a 16-h growth assay. ATc, anhydrotetracycline induction; Uninduced, control. The designation “Empty” (no guide) acts as a control, indicating that the growth defect is not due to ATc administration. With the exception of *groEL* and *rpoC*, the knockdown of most essential genes caused a growth defect, as expected.

**FIG 4:**
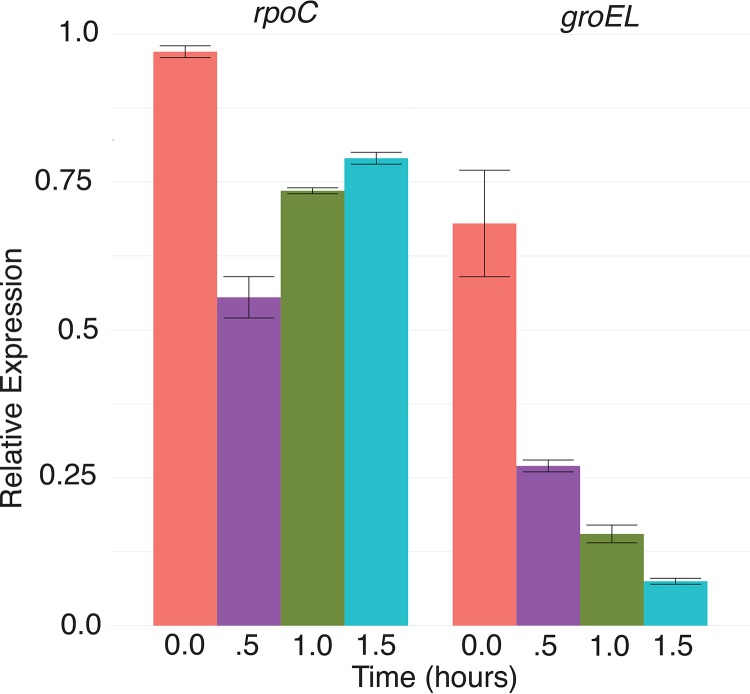
Relative expression levels of *rpoC* and *groEL*. mRNA levels of *rpoC* and *groEL* were measured in knockdown strains over a 1.5-h growth assay. Transcript levels were normalized to the control strain at each time point.

We evaluated the functional utility of GuideFinder guides by random assessment of essential gene knockdown in S. aureus and S. epidermidis, focusing on CRISPRi as a potential application. With the exceptions of *groEl* and *rpoC*, all of the guides showed effective knockdown, which manifested as growth defects (Fig. 3). Further investigation measuring transcription of the locus using quantitative PCR (qPCR) showed that the guide targeting *rpoC* did not reduce the level of transcription (highlighting the value of predicting and testing multiple guides). *groEL* was effectively targeted, but either the gene was nonessential under our tested conditions or residual transcript might have been rescuing cell function (Fig. 4). Thus, our GuideFinder parameters have been used for successful gene knockdown and therefore represent rational design constraints. Overall, these results highlight the utility of the GuideFinder program to create functional guides—demonstrated by functional testing of essential genes—and underscore the need for continued investigation of guide design for improved targeting efficacy in bacterial species. With customizable, user-defined design parameters and access to program source code, users are able to adjust guide selection as this information becomes available.

### Multiguide design.

GuideFinder is also capable of designing guides for multiguide targeting, which may improve the efficacy of knockdown. Aside from the fact that overlapping guides have been shown to reduce knockdown efficiency, very little is known about the impact of the distance between dual targeting guides on gene knockdown in bacteria (6). However, it is plausible that the footprint of the Cas9 protein may influence the ability of two nearby guides to target simultaneously. For this reason and to allow flexibility as new information becomes available, GuideFinder allows users to set a minimum distance threshold that guides selected for dual knockdown must meet. As expected, paired-guide creation—including a 100-bp distance-between-guides threshold—is feasible for fewer genes than single guide creation, owing to the fact that some genes may produce only a single suitable guide or may produce guides that are located in close proximity to each other (Fig. 2C).

**FIG 5:**
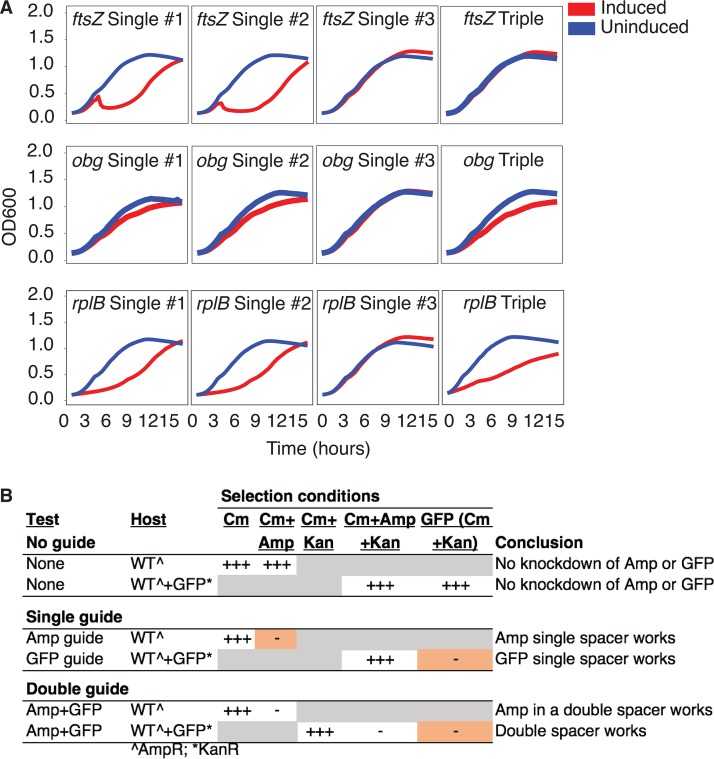
Gene knockdown with multiplex guides. (A) Essential genes were targeted for knockdown in S. aureus using single and triplet guides. Growth curves were created from absorbance measurements over a 16-h growth assay. Triple guide knockdowns contain each of the three single guides targeting as a group. (B) Double tandem guides can simultaneously repress two genes in E. coli. None, one, or two guides were tested, each targeting a chromosomal ampicillin resistance gene and/or a plasmid-borne GFP gene. Drug susceptibilities of the host are indicated. “+++” indicates robust colony growth or GFP signal; “-” indicates that no colonies and no GFP signals were observed. Orange shading indicates critical data. WT, wild type.

Nonetheless, we note that the molecular dynamics of using multiple guides for multiplex targeting within the same gene is more complex than simply accounting for steric hindrance. We tested the hypothesis that the targeting of multiple guides to the same essential gene within one cell would lead to a level of growth defect that is equal to or greater that occurring with a single targeting guide. For three essential S. aureus genes—*ftsZ*, *obg*, and *rplB*—the three guides closest to the TSS were chosen for single-guide and triple-guide knockdown (i.e., for simultaneous targeting). For each gene, the use of the third single guide—that furthest from the TSS—did not result in a growth defect, indicating that it was a nonfunctional guide or that the result represented rescue of gene function due to partial knockdown. For all genes, use of the first two single guides resulted in a growth defect. In the case of *obg* and *rplB*, the triple-targeting construct—in which all three guides were expressed in the same cell—resulted in growth defects similar to those seen with each of the single guides. However, for *ftsZ*, the triple-targeting construct behaved similarly to the nonfunctional third guide, resulting in the absence of a growth defect (Fig. 5A). Overall, the results of the triple-guide targeting experiments were promising but were strongly suggestive of guide-specific and gene-specific effects, representing an area to be further investigated.

On the other hand, targeting separate genes simultaneously could be very effective in double knockdowns. We observed efficient repression of two distinct genes when we tested dual-knockdown efficacy against chromosomal (ampicillin [Amp] resistance) and plasmid (green fluorescent protein [GFP]) targets (Fig. 5B). While the widespread utility of targeting of a single gene with multiple guides will be further investigated, we demonstrated its feasibility and value in double knockdowns. Targeting two different genes simultaneously has significant value for identifying gene-gene interactions, particularly under large-scale conditions, given the facility of CRISPRi.

### Conclusions.

As the first user-friendly, pan-bacterial automated program suitable for large-scale guide selection, this guide finder program is capable of designing guides for any number of genes for any annotated bacterial genome. GuideFinder provides users with a ready-to-use list of designed guides without the need for gene-by-gene score comparison or additional filtering. In this way, the utility of GuideFinder lies in its ability not only to design guides in a large-scale format, including guide multiplexing, but also to provide users with the most suitable guide(s) for each input gene, according to the parameters that they defined. By enabling high-quality, large-scale guide selection for any bacterial genome, GuideFinder improves access to high-throughput studies of bacterial gene function, including genomewide CRISPRi and CRISPRa studies.

## MATERIALS AND METHODS

### GuideFinder implementation.

GuideFinder is written in the R programming language and is available free to use. GuideFinder was written such that it can be used to find guides for both complete and draft genomes, with the recognition that many users may not have a complete genome for the organism of interest. The workflow of the program, including inputs and outputs, is outlined in Fig. 1.

### Inputs and outputs.

**(i) Inputs.** GuideFinder is capable of designing guides for both complete and draft genomes, although the inputs differ slightly.

### Complete genome.

For complete genomes, users simply supply the GenBank accession number and FASTA file.

### Draft genome.

Given the variable organization and notation of draft genomes, annotated draft genome files must be preprocessed prior to inputting into the program. Utilizing the supplied preprocessing script, multisequence FASTA files (e.g., FASTA files containing sequence information for multiple contigs) must be concatenated into a single sequence, with the addition of a series of N’s between contigs. The coordinates of the coding sequences are then identified by aligning the coding sequences against the concatenated FASTA file using BLAST and adjusted to the format required by the main GuideFinder script (i.e., the smaller coordinate, designated the “start” coordinate). These coordinates are then inputted into the main script, along with the single-sequence FASTA file.

**(ii) Outputs.** There are two main outputs of the GuideFinder program: top hits and paired guides lists. Intermediate outputs, such as a list of all possible unfiltered guides, are also made available to the user for reference.

### Top hits list.

The top hits list is a list of guides preferentially selected based on their proximity to the transcription start site. The maximum number of guides supplied per gene is set by the user.

### Paired guides list.

The paired guides list is a list of guide pairs designed to doubly target the same gene in the same cell to increase targeting efficiency. Suitable guide pairs are selected on the basis of the distance between the guides, a parameter set by the user.

### Program Workflow.

**(i) Coordinate identification.** The identification of gene start and end coordinates is the first step in the GuideFinder workflow, and the methods differ slightly for complete versus draft genomes. For complete genomes, the script reads in the annotated genome file containing the gene coordinates and modifies the coordinates to include the putative promoter region. For draft genomes, the coordinates—identified during preprocessing—are directly inputted into the program and modified to include the putative promoter region.

**(ii) Coding and promoter sequence retrieval.** The gene start and end coordinates are used to retrieve the coding and putative promoter sequences from the FASTA file.

**(iii) Guide creation.** Searching within the promoter and gene body, the program identifies NGG PAM sites and utilizes the sequences around each site to create three guides (of lengths 20 bp, 21 bp, and 22 bp) per PAM site. The selection of various guide lengths increases the number of potential guides, many of which are lost to filtering, as described below.

**(iv) Guide filtering.** Guides are filtered according to default and user-defined parameters. By default, the program removes any guides that contain a homopolymer run of A’s or T’s and guides of inadequate length (<20 bp). A user-set threshold is used for filtering based on the maximum distance from the start site, as the targets closest to the transcriptional start site are the ones most likely to disrupt gene function. Guides can be optionally filtered user-set “bad seed” or restriction enzyme sequences and used to minimize off-target effects. For off-target filtering, the first 12 nucleotides (nt) closest to and including the PAM site for each guide are aligned to the FASTA file, and guides that correspond to two or more locations in the genome are discarded. While the sequence consisting of the first 12 nt of the PAM sequence—the seed sequence—represents an established parameter for importance in off-target searching (25), off-target prediction should be experimentally validated for each bacterial species tested prior to large-scale guide design, as differences between species likely exist.

**(v) Final guide selection.** For each PAM site, the program selects the guide of the greatest length that meets the GC minimum set by the user. From these guides, two final guide lists are created, i.e., top hits and paired guides lists, which provide guides and guide pairs suitable for single-gene and dual-gene knockdown, respectively.

**(vi) Iteration.** The program identifies genes that did not produce any guides with the primary parameters. Users have the option to lower these thresholds and re-run these genes through the program to identify additional guides. Users can elect to reduce the GC minimum, increase the maximum guide distance from the transcription start site, retain guides that contain homopolymers, and relax off-target searching. Users can relax each of these guide design constrains individually or in combination.

### Knockdown strain creation.

For both species, knockdown strains were created as follows. For single-guide experiments, a single guide was designed by the GuideFinder program for targeting each gene. For triple-guide targeting (i.e., using three guides expressed in the same cell), the top three guides closest to the TSS were chosen for targeting each gene. The single-guide or triple-guide construct was ligated into our custom CRISPR/dCas9 shuttle vector. Our CRISPR/dCas9 shuttle vector includes all of the necessary components for CRISPRi, including dCas9 (derived from pDB114dCas9 [26]) under the control of an anhydrotetracycline (ATc)-inducible promoter (derived from pRAB11 [27]), dCas9 handle (CRISPR RNA [crRNA] and *trans*-activating small RNA [tracrRNA] fusion, custom designed), and a chloramphenicol (Cm) resistance maker (for selection). The triple-guide targeting vector is a modified version of our CRISPR/dCas9 single vector that enables insertions of multiple guides.

The shuttle vectors containing the proper targeting guides were transformed into E. coli, and the resultant colonies were screened for the guide sequence. A single positive-testing colony was grown in Trypticase soy broth (TSB) with chloramphenicol (TSM/Cm) overnight, and, using a QIAprep Spin Miniprep kit, plasmids were isolated from E. coli and transformed into the staphylococcal species of interest. For S. aureus, plasmids were transformed into competent S. aureus RN4220 cells via electroporation. For S. epidermidis, phagemid transfer was utilized to incorporate the plasmid into S. epidermidis strain Tu3298, according to a protocol previously described elsewhere (28).

For multigene targeting, using host strain E. coli HME63, a gift from Donald Court of the NIH which possess an ampicillin (Amp) resistance gene (CRISPRi target 1), we performed transformations with a plasmid bearing a kanamycin resistance gene (Kan) and a constitutive GFP gene (CRISPRi target 2). We then cloned either single guides targeting each gene individually or double guides targeting both genes simultaneously into our modified dCas9 vector. Six independent transformations were performed, the results were plated onto the appropriate selective agar plates, and CFU counts were performed for the Amp guide group. Single colonies from the GFP guide group were grown, and the fluorescence of the cultures was measured with a Cytation 3 imaging reader (BioTek).

### Growth assays.

Growth assays were performed to assess knockdown of essential genes. The growth assays were performed in both S. aureus and S. epidermidis as follows. A single colony of each knockdown strain was grown overnight in TSB containing chloramphenicol. The overnight culture was diluted to an optical density (OD) of 0.05 in TSB/Cm, grown to an OD of 0.5, and diluted again at the start of the assay to an OD of 0.05 with TSB/Cm (control group) or TSB/Cm–0.1 μM anhydrotetracycline (inducer, experimental group). The cultures were grown for 16 h, with OD measurements taken each half-hour to construct a growth curve for each knockdown strain. For each strain, the induced/experimental group growth curve was compared to the uninduced/control group curve. Knockdown of most of the essential genes resulted in a severe growth defect, as expected. The knockdown of two genes, *groEL* and *rpoc*, did not result in the expected growth defect, and we investigated the ability of each guide to reduce transcript levels.

### Measuring transcript levels.

In S. aureus, we measured transcript levels of *groEL* and *rpoc* growing in liquid media to determine if the selected guide was capable of reducing transcript levels. A single colony of each *groEL* and *rpoc* knockdown S. aureus strain was grown overnight in TSB/Cm at 37°C with shaking. The overnight culture was back-diluted to an OD of 0.05 and was grown at 37°C until an optical density at 600 nm (OD_600_) of 0.5 was reached. The culture was back-diluted again to an OD of 0.05 with TSB containing chloramphenicol and 0.1 μM anhydrotetracycline and was grown for 1.5 h; readings were taken at time points throughput the assay (h 0, 0.5, 1, and 1.5). An aliquot taken at each time point was mixed with 2 volumes of RNA Protect and incubated for 5 min at room temperature. The aliquot was spun down, and the supernatant was decanted and stored at −20°C until RNA extraction. RNA from the four time points was extracted according to the protocol for an RNeasy Plus kit, with an added enzymatic digestion step performed using lysozyme and lysostaphin for lysis of the Gram-positive species S. aureus. RNA was reversed transcribed to create cDNA by the use of a High-Capacity cDNA reverse transcription kit (Applied Biosystems), according to provided instructions. Quantitative PCR (qPCR) was performed using PowerUp SYBR green master mix (Applied Biosystems) in conjunction with gene-specific primers. Primers amplifying the *ftsZ* gene were used as an internal control, and nontemplate controls were included. Duplicate qPCR reactions were performed for each assay as a technical replicate.

The genomes used for draft genome analysis were obtained from PATRIC. The strain used and the genome identifier (ID) numbers are as follows: Micrococcus luteus ATCC 12698 (Genome ID 1270.61), Micrococcus luteus O’Kane (Genome ID 1270.50), Staphylococcus aureus WBG10049 (Genome ID 585160.3), Staphylococcus aureus SA14-296 (Genome ID 46170.233), Staphylococcus epidermidis NLAE-zl-G239 (Genome ID 1282.2004), and Staphylococcus epidermidis FDAARGOS_83 (Genome ID 1282.1163).

### Availability of and requirements for GuideFinder.

Details of the availability of and requirements for GuideFinder are as follows: project name, GuideFinder; project home page, https://github.com/ohlab/Guide-Finder; operating system(s), Mac, Windows; programming language, R. Other requirements are as follows: license, none; restrictions for use by nonacademics, none.

### Data availability.

The genomes used for complete genome analysis were obtained from NCBI. The accession numbers for each strain are as follows: for Lactobacillus brevis, GenBank accession no. CP000416.1; for Lactobacillus jensenii, GenBank accession no. CP018809.1; for Staphylococcus epidermidis, GenBank accession no. AE015929.1; for Staphylococcus aureus, GenBank accession no. CP000253.1; for Rhizobium leguminosarum, GenBank accession no. CP007045.1; for Pseudomonas aeruginosa, GenBank accession no. AE004091.2; for Mycobacterium tuberculosis, GenBank accession no. AL123456.3; for Micrococcus luteus, GenBank accession no. CP001628.1; for Streptomyces scabiei, GenBank accession no. FN554889.1 .
